# Transcriptomic Effects of Oclacitinib and Prednisolone in an Acute IgE-Mediated Experimental Model of Canine Atopic Dermatitis

**DOI:** 10.3390/vetsci13070676

**Published:** 2026-07-13

**Authors:** Renato Leon, Amanda Blubaugh, Haley Starr, Frane Banovic

**Affiliations:** College of Veterinary Medicine, University of Georgia, Athens, GA 30602, USA; rgl84554@uga.edu (R.L.); alblub@uga.edu (A.B.); haley.starr@uga.edu (H.S.)

**Keywords:** canine, transcriptome, atopic, RNA sequencing, immunoglobulin-E, late phase reactions

## Abstract

Intradermal injections of anti-canine immunoglobulin E (IgE) in healthy dogs have been utilized in preclinical drug testing to evaluate the efficacy of anti-allergic drugs used to treat canine atopic dermatitis (AD). However, the molecular effects of established canine anti-allergic drugs on this acute canine IgE-mediated atopic model remain largely uninvestigated. The objective of this study was to characterize the effect of proactive oclacitinib and prednisolone treatments on the immune and skin barrier transcriptome of IgE-mediated late-phase reactions (LPRs) in an acute model of canine AD. Sixteen healthy adult research-bred beagles were randomized to receive either oclacitinib or prednisolone orally for six days, followed by an intradermal anti-canine IgE injection. Biopsies were collected 24 h post-injection for RNA isolation and sequencing; previously analyzed transcriptomes (healthy skin, saline-injected skin, IgE lesions without drug modulation) from the same colony of dogs served as controls. Administration of prednisolone and oclacitinib prior to intradermal anti-IgE injections reduced the number of differentially expressed genes (DEGs) in 24 h samples to 1251 and 1471, respectively. Both treatments resulted in a decrease in expression of several significantly upregulated T helper-(Th)1 (e.g., *MX1*, *OAS1*, *STAT1*), Th2 (e.g., *CCL13*, *CCL8*, *IL13RA1*, *IL-33*, *IL5RA*, *OSM*), chemokine and receptor (e.g., *CCL19*, *CCL2*, *CCL3*, *CCR1*, *CCR3*) genes in comparison to the untreated IgE-mediated lesions. Interestingly, only prednisolone treatment significantly reduced *IL-13* upregulation, an important gene in the Th2 immune response. In conclusion, both prednisolone and oclacitinib reduced the transcriptomic changes observed in the acute lesions of the canine IgE-induced atopic dermatitis model, with prednisolone inducing a broader inhibitory immune response.

## Simple Summary

Atopic dermatitis is the most common inflammatory skin condition affecting dogs and humans. Experimental models of AD are crucial to study the disease and advance the development of novel therapeutic agents. Intradermal injections of anti-canine immunoglobulin E (IgE) have been used in healthy dogs to induce acute lesions that replicate the itch and inflammation observed in spontaneously occurring AD. However, the molecular effect of established canine anti-allergic drugs in this acute canine IgE atopic model remains largely uninvestigated. This study utilized RNA sequencing to characterize the transcriptomic effect of anti-allergic drugs oclacitinib and prednisolone on acute IgE-induced lesions in sixteen healthy adult research-bred beagle dogs. Oclacitinib and prednisolone treatments both modulated the immune and skin barrier transcriptome in the experimental acute IgE-mediated atopic model, with prednisolone exerting a broader modulatory response.

## 1. Introduction

Atopic dermatitis (AD) is a common, chronic and frequently relapsing inflammatory skin disease. The pathogenesis of canine AD is multifactorial and remains incompletely understood. It involves complex interactions between environmental and genetic factors, impaired epithelial barrier integrity, immune dysfunction, initiation of the itch–scratch cycle and alterations in the cutaneous microbiome [1]. These factors collectively contribute to the initiation and maintenance of AD skin lesions. Increased pruritus in affected dogs results in significant sleep disruption and diminished quality of life, impacting both canine patients and their owners [1].

Inducible canine AD models (e.g., epicutaneously sensitized house dust mite model, acute canine IgE-induced lesions, IL-31-induced pruritic model) have been used extensively in experimental research to advance the development of novel medications that inhibit immune and itch-inducing pathways in AD [2,3,4,5,6,7,8,9]. In one of the inducible acute canine AD models, anti-canine IgE antibodies are injected intradermally into healthy dogs to experimentally induce late-phase reactions (LPRs) that histologically resemble the inflammatory cell infiltration observed in naturally occurring canine AD at 6 and 24 h [10,11]. This inducible IgE-mediated model has been utilized as a preclinical screening tool to assess the clinical and histopathological anti-inflammatory effect of anti-allergic drugs used to treat canine AD [3,12,13]. A recent transcriptomic study of RNA-sequencing (RNA-seq) data revealed that acute IgE-mediated LPR lesions in healthy dogs exhibit significant upregulation of proinflammatory, Th1/IFNγ, and Th2 (e.g., *IL4R*, *IL-5*, *IL-13*, *IL-33*, *POSTN*, *CCL17*, *CCL24*) genes. Notably, shared differentially expressed genes (DEGs) in canine LPR lesions (6 h and 24 h post-anti IgE injections) demonstrated a significant, moderate, positive correlation with spontaneous human AD skin lesions [14].

Transcriptomic studies using gene expression microarrays and RNA-seq have demonstrated that immunomodulatory treatments (e.g., topical glucocorticoids, cyclosporine, biologics, JAK inhibitors) normalize cutaneous gene expression in human AD lesional skin, shifting expression levels of many of the genes (immune and skin barrier markers) dysregulated in active lesions toward levels found in nonlesional AD skin [15,16]. In contrast to human AD, relatively few studies have characterized the molecular effects of available drug treatments in either inducible canine AD models or spontaneous canine AD. In a single RNA-seq study, lokivetmab failed to attenuate the upregulation of cytokines *IL-6*, *IL-9*, *IL-13*, *IL-33*, *CCL17*, and *CCL22* in experimental acute HDM-induced canine AD lesions, identifying these as possible targets for inhibition that might complement the action of lokivetmab in the treatment of canine AD [2]. Furthermore, an experimental pilot study evaluating acute atopic IgE-mediated LPRs in five dogs reported that short-term systemic prednisolone treatment suppressed leukocyte influx while increasing the expression of selected pro-allergic/inflammatory genes (e.g., *IL-13*, *CCL2*, *CCL5*, *CCL17*) by reverse transcription quantitative PCR (RT-qPCR) [11]. To the best of our knowledge, no other studies have evaluated drug-induced modulation of the global transcriptome in skin lesions of any inducible preclinical models of canine AD.

As advances in our understanding of AD drive the development of specialized treatments that specifically target molecules involved in AD pathogenesis, it is increasingly important to understand the molecular changes induced by immunomodulators in inducible preclinical AD models and spontaneous canine AD. Given the current limited understanding of immunomodulation in acute canine IgE-induced lesions, this study aimed to characterize the effect of systemic oclacitinib (JAK1/2 inhibitor) and prednisolone (glucocorticoid) treatments on the inflammatory and skin barrier transcriptome of experimental acute IgE-induced canine skin lesions using RNA-seq in healthy dogs. Oral oclacitinib and prednisolone were selected for evaluation due to their widespread availability, established efficacy and recommendation as systemic treatment options for the management of canine AD by international treatment guidelines. Additionally, this study provided an opportunity to reassess the limited immunomodulatory effects of prednisolone reported in a previous pilot study of IgE-mediated LPRs in five dogs [11,17,18].

## 2. Materials and Methods

### 2.1. Study Population

Sixteen healthy adult male research-bred beagles with no previous history of pruritus or skin disease were enrolled in this study. All participants were between one to five years of age at the time of the study. The dogs were housed in kennels in university laboratory animal facilities under standard conditions. No systemic immunomodulating medications were provided to the dogs for 12 weeks before study enrollment to eliminate any potential influence from other drugs. All aspects of this study were approved and conducted in accordance with the Institutional Animal Care and Use Committee (IACUC; protocol code A2017 08-021-Y3-A5). Eight dogs were assigned to each treatment group (oral prednisolone or oclacitinib); this sample size was deemed sufficient to achieve 80% power to detect a significant 1.5-fold difference in mRNA transcription values between treatment groups and untreated controls using ssizeRNA [19].

### 2.2. Interventions and Randomizations

All dogs were initially randomized to receive either oclacitinib (*n* = 8; Apoquel, Zoetis Inc., New York, NY, USA) or prednisolone (*n* = 8; PrednisTab, Lloyd, Shenandoah, IA, USA) using Prism 10.0 (GraphPad Software, La Jolla, CA, USA). Based on the assigned treatment groups, dogs received oclacitinib at 0.4 to 0.6 mg/kg or prednisolone at 0.5 mg/kg orally twice daily for five consecutive days, followed by a single morning dose on day 6, for a total of 11 oral drug treatments per dog. The dosages and frequency of oclacitinib and prednisolone administration were chosen based on manufacturer recommendations, a previous pilot study investigating the effect of prednisolone on atopic acute IgE-mediated LPRs, and pharmacokinetic data indicating when the steady state of each drug is achieved in circulation [11,20,21].

The atopic acute IgE-mediated LPRs were induced immediately after oral drug administration as previously described [11,14]. Briefly, a small (about 10 × 10 cm) area for the injection of substances was clipped on the right lateral thoracic region; all injection site clippings were performed ≥48 h before each injection to avoid the occurrence of any localized skin reaction or irritation in concurrence with the administration and evaluation of the substances. Intradermal injections of 0.05 mL anti-canine-IgE polyclonal antibodies (0.08 mg/mL, goat anti-canine IgE AHP946, Bio-Rad Laboratories, Inc., Hercules, CA, USA) were administered to all dogs. The order of injections was randomized for each dog using statistical software (GraphPad Software), and the investigator (XX) was blinded during intradermal injection administration and evaluations.

### 2.3. Clinical Lesion Assessment and Skin Biopsy Collections

Clinical scoring of the injection sites was performed by a blinded investigator (XX), who measured the LPR score at 24 h post-injection [11,14]. To assess LPRs, erythema (E) and induration (firmness, F) were scored from 1 to 3 (1, no erythema/flaccid wheal; 2, weak erythema/moderate induration; 3, strong erythema/firm induration). LPR scores were then calculated as follows: LPR = E × F. At 24 h post-injection, dogs were sedated intravenously with medetomidine (Domitor, Pfizer, Exton, PA, USA), and 8 mm skin biopsy samples were collected from IgE-mediated cutaneous reactions; all biopsies were immediately immersed in RNAlater (Thermo Fisher Scientific, Vilnius, Lithuania) and stored at −80 °C until RNA extraction.

### 2.4. Control Data

Clinical evaluation scores and transcriptomic data from a previous study characterizing the experimental atopic acute IgE-mediated LPR model on the same beagle colony (healthy adult male research-bred beagles with no previous history of pruritus or skin disease), conducted in the absence of immunomodulatory drugs, were obtained and reanalyzed as controls (healthy skin, saline-injected skin, IgE lesions without drug modulation) [14]. Briefly, intradermal injections of 0.05 mL anti-canine-IgE polyclonal antibodies (0.08 mg/mL, goat anti-canine IgE AHP946, Bio-Rad Laboratories, Inc.) and 0.05 mL phosphate-buffered saline (PBS, diluent, negative control; Sigma-Aldrich, St. Louis, MO, USA) were administered to the dogs. Clinical evaluation and biopsy collections were performed at 24 h post-challenge. Baseline healthy skin biopsies were collected seven days prior to anti-canine-IgE and saline injections [14].

### 2.5. Statistical Analysis of Clinical Lesion Assessment

Clinical evaluation data (LPR scores) for each group (saline-injected skin, IgE lesions without drug modulation, IgE lesions on prednisolone, IgE lesions on oclacitinib) were compared using a nonparametric one-way ANOVA (Kruskal–Wallis test) with *p* < 0.05 as the significance threshold.

### 2.6. RNA Extraction and RNA-Sequencing

Total mRNA was extracted from all drug (prednisolone or oclacitinib)-modulated IgE-mediated cutaneous reaction biopsies using the Qiagen miRNAeasy Mini Kit (Qiagen, Venlo, The Netherlands) according to the manufacturer’s specifications. A total of 16 RNA samples (*n* = 8 per group) with a 260/280 ratio of ~1.8–2.0 and an RNA integrity number (RIN) greater than 7 were selected for library preparation and sequencing. Samples were sequenced with 150 paired-end base pairs using the NovaSeq 6000 system (Illumina, San Diego, CA, USA).

### 2.7. Transcriptomic Analysis

The quality of raw sequence data for all groups (healthy untreated skin, IgE-mediated LPRs at 24 h, saline injections group at 24 h without drug modulation, and IgE-mediated LPRs at 24 h after prednisolone and oclacitinib) was first evaluated using fastQC [22]. After quality control, all raw reads were mapped to the canine genome (canFam6) using the STAR aligner [23]. Alignment quality was then assessed using Qualimap, and featureCounts was used to quantify gene expression [24,25]. Normalization of RNA-seq data and differential expression (DE) analysis between different conditions compared to healthy skin (saline-injected skin, IgE lesions without drug modulation, IgE lesions on prednisolone, IgE lesions on oclacitinib) and IgE-mediated LPRs (IgE lesions on prednisolone, IgE lesions on oclacitinib) was performed using the R package DESeq2, which utilizes a negative binomial generalized linear model to determine differential expression and incorporates empirical Bayes methods to perform shrinkage estimation (Supplementary Table S1) [26]. Differentially expressed genes were defined as those with a false discovery rate (FDR) < 0.05 and an absolute fold change (FC) > 1.5.

### 2.8. Functional and Biological Pathway Analysis

An unbiased comparison of gene set variation between groups was performed using the nonparametric, unsupervised Gene Set Variation Analysis (GSVA) method, with a focus on the Th1, Th2, Th17, and Th22 pathways [14,27]. Differential expression (FDR < 0.05) of distinct immune gene sets between groups was then assessed using limma, which combines a linear model approach with empirical Bayes methods [28].

Functional enrichment analysis of downregulated DEGs (FC < 1.5 and FDR < 0.05) in IgE lesions treated with oclacitinib or prednisolone compared to untreated IgE lesions was performed using the Process Networks tool from the MetaCore platform (MetaCore, Clarivate Analytics, London, UK) [29]. Network enrichment scores were considered significant at FDR < 0.05.

## 3. Results

### 3.1. Late Phase Reaction Scores

**Figure 1:**
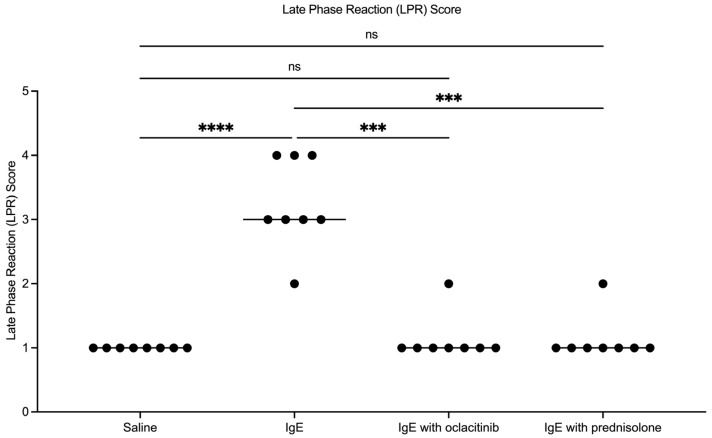
Anti-canine IgE injections induced a strong increase in late phase reactions (LPRs) at 24 h compared to saline control. Oclacitinib and prednisolone treatments significantly reduced LPRs in IgE lesions compared to the untreated group. No statistical difference in LPRs was observed between drug-treated IgE lesions and saline-injected skin. Each dot represents a dog. Medians are included for each group. Significance: ns = non-significant; *** *p*-adj < 0.001; **** *p*-adj < 0.0001.

As previously reported, the evaluation of LPR scores revealed that anti-canine-IgE injections induced significantly greater LPRs in healthy skin than saline injections at 24 h (Figure 1; *p* < 0.0001). Modulation of IgE-mediated lesions with oclacitinib and prednisolone significantly reduced the LPR scores of acute lesions induced by anti-canine-IgE injections at 24 h (*p* = 0.0003). No significant difference in LPR scores was detected between saline-injected skin and drug (prednisolone and oclacitinib)-modulated IgE-mediated lesions at 24 h.

### 3.2. Effect of Oclacitinib and Prednisolone Treatments on the Transcriptome of IgE-Mediated Late Phase Reactions (LPRs)

**Figure 2:**
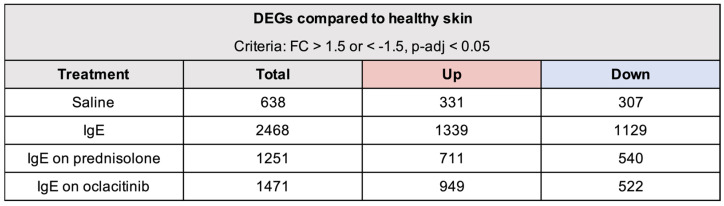
Number of differentially expressed genes (DEGs) across different treatment groups at 24 h relative to healthy skin (*n* = 8 per group). Saline-injected skin (negative) and IgE-mediated lesions without drug modulation (positive) served as controls. Anti-canine IgE injections induced a four-fold increase in total DEGs compared to the saline negative control. Oclacitinib and prednisolone treatments reduced the total number of DEGs in IgE-mediated lesions by 40% and 49%, respectively. Differential expression was defined by absolute FC > 1.5 and FDR < 0.05. Color corresponds to upregulated (red) or downregulated (blue) DEGs.

RNA-seq analysis was used to profile saline-injected skin, IgE lesions without drug modulation, IgE lesions on prednisolone, and IgE lesions on oclacitinib at 24 h compared to respective healthy skin (Figure 2). Using criteria of absolute FC  > 1.5 and FDR < 0.05 to define DEGs, we identified 2468 DEGs (1139 upregulated and 1129 downregulated) in IgE-mediated LPRs at 24 h, a 4-fold DEG increase compared with the saline negative control, which induced 638 DEGs (331 upregulated and 307 downregulated). Immunomodulatory treatment with both oclacitinib and prednisolone significantly reduced the number of DEGs in IgE-mediated LPRs at 24 h. Prednisolone potently reduced the number of DEGs to 1251 (711 upregulated and 540 downregulated), whereas oclacitinib treatment resulted in a total of 1471 DEGs (949 upregulated and 522 downregulated) in IgE-mediated LPRs at 24 h.

Principal component analysis (PCA) was used to visualize differences in gene expression profiles between groups (Supplementary Figure S1). As previously reported, the fitted ellipses of saline-injected skin and IgE lesions without drug modulation compared to healthy skin showed a clear separation in the transcriptomic profiles of these groups at 24 h. Treatments with oclacitinib and prednisolone reduced the overall molecular changes induced by intradermal anti-IgE injections without drug modulation, shifting the transcriptome closer to the state observed in healthy skin samples.

**Figure 3:**
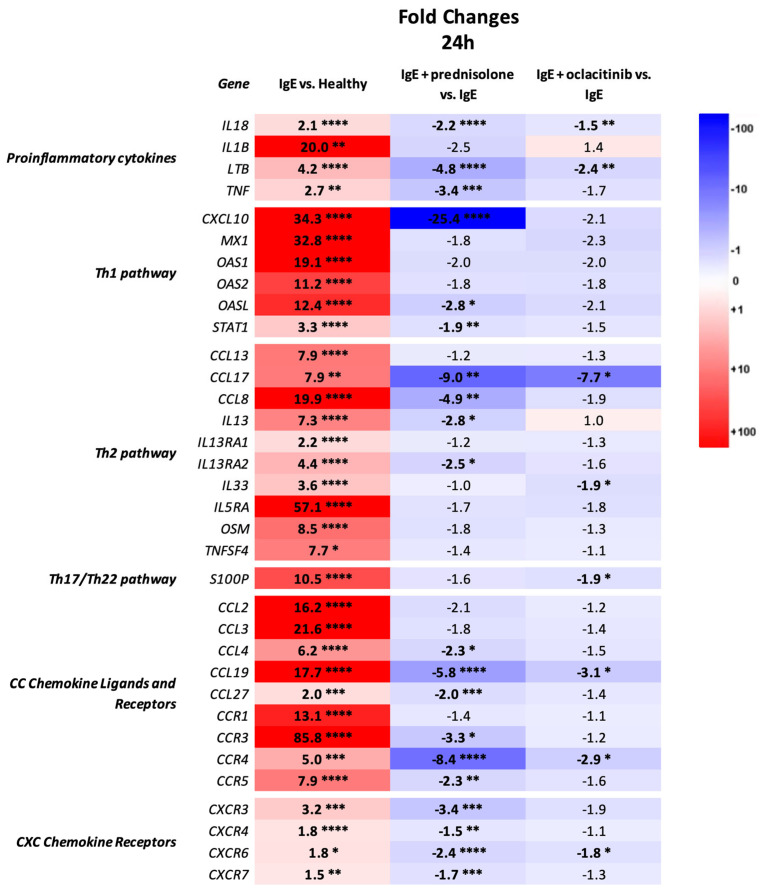
Expression of selected relevant proinflammatory, immune, and skin barrier genes in IgE lesions without drug modulation versus healthy skin, IgE lesions on prednisolone versus IgE lesions without drug modulation, and IgE lesions on oclacitinib versus IgE lesions without drug modulation at 24 h following intradermal injections (*n* = 8 per group). Anti-canine IgE injections induced the upregulation of several Th1, Th2, and pro-inflammatory cytokines compared to healthy skin. Prednisolone exerted a stronger modulation of immune and skin barrier markers dysregulated in IgE-mediated lesions than oclacitinib. Genes are grouped by their function or family. Color corresponds to increased (red) or decreased (blue) gene expression. Statistically significant fold changes (FC) are bolded and marked with asterisks corresponding to their significance value. Significance: * *p*-adj < 0.05; ** *p*-adj < 0.01; *** *p*-adj < 0.001; **** *p*-adj < 0.0001.

Consistent with prior observations, anti-canine IgE injections significantly upregulated several important proinflammatory and immune markers related to Th1/IFNγ and Th2 (e.g., *IL4R*, *IL-5*, *IL-13*, *IL-33*, *POSTN*, *CCL17*, *CCL24*) pathways compared to healthy skin (Figure 3). Molecular profiling revealed stronger prednisolone-mediated regulation of IgE-mediated immune responses than oclacitinib in this acute atopic model. Both drug treatments, prednisolone and oclacitinib, significantly reduced the expression of several genes upregulated in IgE-mediated LPRs at 24 h, including proinflammatory (*IL-18* and *LTB*), Th2 (*CCL17*), and chemokine (*CCL19*, *CCR4*, *CXCR6*) markers, with prednisolone inducing a more robust downregulation of these genes than oclacitinib. Furthermore, prednisolone treatment significantly downregulated additional important proinflammatory markers (TNF-α), Th1 markers (*CXCL10*, *OASL*, *STAT1*), Th2 markers (*CCL8*, *IL-13*, *IL13RA2*), and chemokine markers (*CCL19*, *CCL27*, *CCR3*, *CCR5*, *CXCR3*, *CXCR4*, *CXCR7*) in IgE-mediated LPRs at 24 h. Although downregulation of these genes was also observed in the oclacitinib group, the effect was smaller and did not reach significance.

To evaluate the molecular effect of anti-allergic drugs prednisolone and oclacitinib on the skin barrier of the IgE-mediated LPRs at 24 h, we analyzed the expression of epidermal barrier differentiation and lipid-associated genes (Supplementary Figure S2) [30,31,32]. Both prednisolone and oclacitinib reversed the significant decreases in barrier differentiation genes of IgE-mediated LPRs at 24 h related to terminal differentiation (*CDSN*, *LCE6A*, *ST14*, *TGM1*) and gap/tight-junctions (*CLDN1*, *CLDN4*, *GJB7*); only prednisolone significantly reversed lipid metabolism/biosynthesis markers (*FADS2*, *FABP3*, *FABP4*, *LPIN1*). Interestingly, prednisolone and oclacitinib showed non-significant opposing effects on *CLDN1* and *LCE6A* markers, respectively. Furthermore, both drug treatments altered the expression of several barrier markers that were not altered in the untreated IgE-mediated LPRs at 24 h compared to healthy skin, including the downregulation of important barrier markers *FLG*, *IVL* and *FADS1* (prednisolone-only), *TGM3* and *CLDN2* (oclacitinib-only), and *DSC1*, *CLDN2*, *CLDN11*, *ALOX12B*, *ELOVL1* and *HMGCS1* (prednisolone and oclacitinib).

### 3.3. Pathway and Enrichment Analysis of Oclacitinib and Prednisolone Treatments on Experimental Atopic Acute IgE-Mediated LPRs

**Figure 4:**
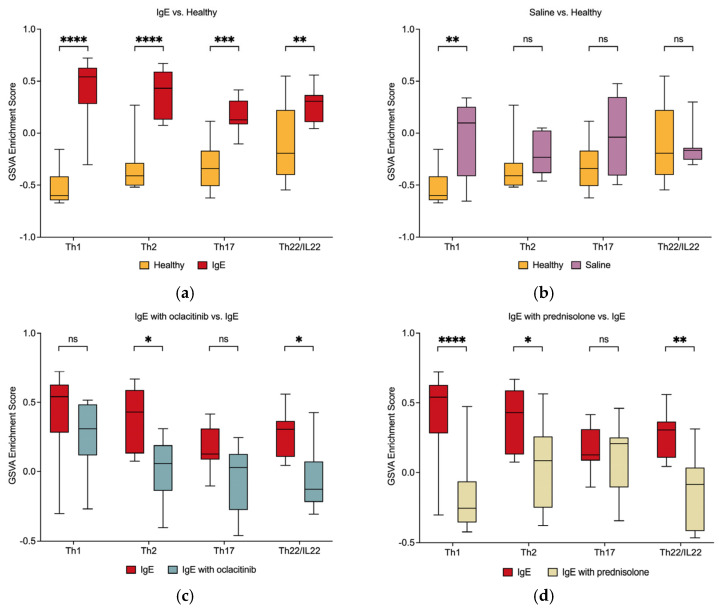
Gene Set Variation Analysis (GSVA) enrichment scores for Th1, Th2, Th17 and Th22/IL22 immune pathways in (**a**) healthy skin versus IgE lesions without drug modulation, (**b**) healthy skin versus saline-injected skin, (**c**) IgE lesions without drug modulation versus IgE lesions on oclacitinib at 24 h, and (**d**) IgE lesions without drug modulation versus IgE lesions on prednisolone at 24 h. Prednisolone treatment significantly downregulated the expression of the Th1, Th2, and Th22/IL22 pathways; oclacitinib only significantly reduced the expression of the Th2 and Th22/IL22 pathways. Medians, quartiles and range are included for each group (*n* = 8 per group). Significance: ns = non-significant; * *p*-adj < 0.05; ** *p*-adj < 0.01; *** *p*-adj < 0.001; **** *p*-adj < 0.00015.

To better understand the impact of prednisolone and oclacitinib treatments on the immune response of IgE-mediated reactions, GSVA was performed using previously published human gene sets for the Th1, Th2, Th17, and Th22/IL22 pathways, which were adjusted to the respective canine orthologs [14,33]. Consistent with the original study findings, re-analysis of the healthy and anti-canine-IgE-injected skin transcriptomes showed a significant upregulation of the Th1 (*p* < 0.0001), Th2 (*p* < 0.0001), Th17 (*p* = 0.0006) and Th22/IL22 (*p* = 0.008) pathways in untreated acute IgE-mediated LPRs at 24 h compared with healthy skin (Figure 4) [14]. Modulation of IgE-mediated LPRs with prednisolone downregulated all four assessed immune pathways compared with untreated IgE lesions, with significant downregulation of the Th1 (*p* < 0.0001), Th2 (*p* = 0.02), and Th22/IL22 (*p* = 0.006) pathways. In contrast, oclacitinib only significantly reduced the Th2 (*p* = 0.03) and Th22/IL22 (*p* = 0.03) pathways.

**Figure 5:**
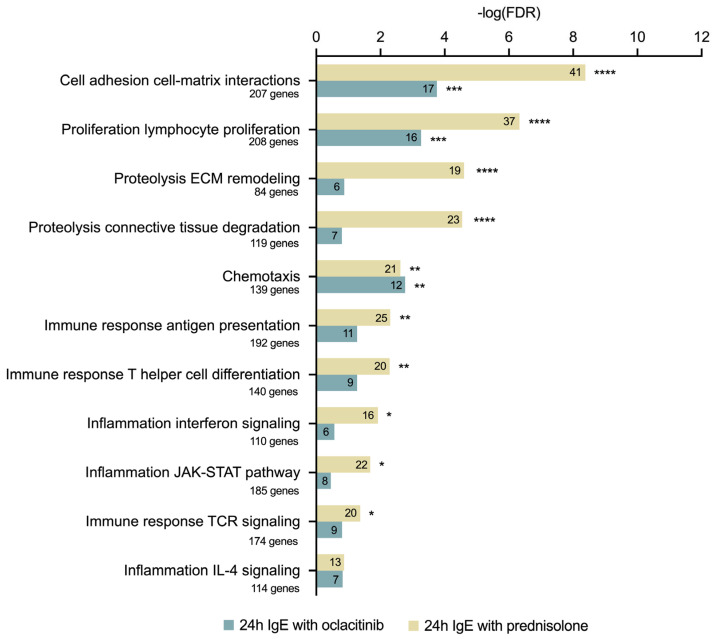
Selected MetaCore process networks for downregulated DEGs (FC < −1.5 and FDR < 0.05) revealed a greater immunomodulatory response by prednisolone treatment compared to oclacitinib on IgE-induced skin lesions compared to untreated lesions at 24 h after intradermal injections. cance: * *p*-adj < 0.05; ** *p*-adj < 0.01; *** *p*-adj < 0.001; **** *p*-adj < 0.0001.

To identify the biological pathways that were differentially altered by drug treatment, we performed MetaCore enrichment analysis of DEGs downregulated by prednisolone and oclacitinib in acute IgE-mediated reactions (Figure 5, Supplementary Table S2). As previously reported, 48 process networks were upregulated in untreated anti-IgE-injected skin at 24 h compared to healthy skin; the top 20 process networks were related to interferon signaling, leukocyte chemotaxis, JAK-STAT signaling, lymphocyte proliferation, antigen presentation, phagocytosis, regulation of cytoskeleton rearrangement, histamine signaling, T-helper cell differentiation, NK-cell cytotoxicity and IL-4 signaling [14]. Downregulated DEGs in IgE-mediated LPRs treated with prednisolone were significantly enriched in 21 process networks, including lymphocyte proliferation and TCR signaling, cell–matrix interactions, leukocyte chemotaxis, antigen presentation, interferon signaling, T-helper cell differentiation, and JAK-STAT signaling. In contrast, oclacitinib-treated IgE-mediated LPRs showed only six significantly enriched process networks at 24 h (e.g., cell–matrix interactions, leukocyte chemotaxis, lymphocyte proliferation, chemotaxis, NK cell cytotoxicity).

## 4. Discussion

In the present study, we aimed to analyze the immunomodulatory effects of the commonly used anti-allergic drugs prednisolone and oclacitinib on the global transcriptome of the experimental canine atopic acute IgE-mediated LPR model in healthy dogs. To the best of the authors’ knowledge, this study is the first to show that prednisolone (glucocorticoid) and oclacitinib (JAK inhibitor) inhibit the global gene expression of inflammatory and immune markers, while also modulating the expression of genes associated with epidermal barrier differentiation and lipid composition in IgE-induced reactions at 24 h.

Historically, the management of canine AD has relied primarily on the administration of systemic and/or topical glucocorticoids (e.g., prednisolone, prednisone, methylprednisolone) [34]. Glucocorticoids are potent broad-acting immunomodulatory and anti-inflammatory agents that regulate transcription by binding to cytoplasmic glucocorticoid receptors. This complex then translocates to the nucleus, where interactions with other transcription factors reduce the expression of proinflammatory mediators and promote anti-inflammatory cytokine production [35,36,37]. In addition to regulating gene expression, glucocorticoids regulate various cellular processes (e.g., carbohydrate metabolism, hormone secretion, neuronal function) within minutes through non-genomic interactions with membrane-bound glucocorticoid receptors [38,39]. Despite glucocorticoid effectiveness in managing acute AD clinical symptoms, long-term glucocorticoid use can lead to increased risk of adverse side effects [34,40]. Studies evaluating the immunomodulatory effects of glucocorticoids on the skin transcriptome in human AD patients have been limited to topical formulations. In a placebo-controlled clinical trial, transcriptome analysis of triamcinolone acetonide cream (0.025%) treatment induced a significant downregulation of several Th1 (e.g., *IL-12*, *IL-12B*, *CXCL10*), Th2 (e.g., *IL-13*, *CCL17*, *CCL18*), and proinflammatory (e.g., *IL1B*) markers in lesional AD skin of moderate-to-severe AD patients [41]. A recent randomized, intra-patient, vehicle-controlled study revealed that administration of triamcinolone acetonide cream (0.5%) twice daily for 72 h induced a significant (*p* < 0.05) downregulation in lesional AD skin of various immune genes, including those involved in the Th1 (*CCR1*, *CXCL9*, *CXCL10*), Th2 (*IL-9*, *IL-13*, *CCL13*, *CD40LG*), Th17 (*IL-19*, *IL-20*, *IL-23A*, *IL36RN*, *CAMP/LL37*), and Th22 pathways (*IL22*, *S100A7*) compared to healthy skin [42]. To the best of the author’s knowledge, there have not been any in-depth evaluations of the effects of systemic and/or topical glucocorticoid use on the cutaneous immunological response in canine AD models and spontaneous canine AD patients. Unexpectedly, a previous evaluation of molecular changes by RT-qPCR in acute atopic IgE-mediated LPRs in five dogs reported that short-term oral prednisolone treatment increased expression of selected pro-allergic/inflammatory genes, including *IL-13*, *CCL2*, *CCL5*, and *CCL17* [11]. In contrast, oral prednisolone treatment in our study produced a strong, broad immunosuppressive and anti-inflammatory response in IgE-mediated LPRs at 24 h, significantly reducing the expression of major pro-inflammatory (e.g., *TNF*, *IL-18*), chemokine and chemokine receptor (e.g., *CCR3*, *CCR4*, *CCL27*), Th1 (e.g., *CXCL10*, *OASL*, *STAT1*), and Th2 (e.g., *CCL17*, *IL-13*, *CCL8*) markers. Our study results align with the immunomodulatory and anti-inflammatory changes observed after topical glucocorticoid treatment in human lesional AD skin [41,42].

The emergence of targeted anti-allergic drugs, including JAK inhibitors (e.g., oclacitinib, ilunocitinib, atinvicitinib) and monoclonal antibodies (e.g., lokivetmab), has provided effective alternatives to glucocorticoids for the treatment of spontaneous canine AD [43]. Oclacitinib, a non-selective JAK inhibitor with higher JAK1 and JAK2 affinity, provides fast-acting relief to canine AD patients by inhibiting proallergic cytokine signaling in the JAK/signal transducer and activator of transcription (JAK/STAT) pathway [44,45,46]. Studies using isolated enzyme systems as well as in vitro human or canine cell line models have demonstrated the efficacy of oclacitinib in reducing the activity of important proinflammatory (*IL-2* and *IL-6*), pro-allergic (*IL-4* and *IL-13*) and pruritic (*IL-31*) cytokines [21,44]. Recently, a study characterizing the effects of abrocitinib, a selective JAK1 inhibitor, in human patients with moderate to severe AD demonstrated clinical symptom improvement and reduced itch in treated patients [16]. At the molecular level, abrocitinib significantly decreased the expression of Th2 (e.g., *CCL17*, *CCL18)* and Th17/22 (e.g., *S100A* proteins, *IL36G*) markers in lesional AD skin compared to baseline levels. Another transcriptomic study evaluating an oral JAK/spleen tyrosine kinase (SYK) inhibitor showed broad immunomodulation by the drug, with RT-qPCR and microarray data showing a significant decrease in the expression of important Th1 (*IFNy*, *CXCL9/CXCL11*, *MX1*), Th2 (*IL4R*, *IL-13*, *CCL13*, *CCL17*, *CCL18*, *CCL22* and *CCL26*), and Th17/22 (lipocalins, *PI3*, *CCL20*, *S100A7*, *S100A8*, *S100A9*, and *IL36G/IL36RN*) genes following treatment of lesional AD skin in human patients [47]. There have been no studies evaluating the modulation of cutaneous immunological responses in spontaneous AD dogs and models treated with any veterinary JAK inhibitor. In our experimental acute canine IgE model, systemic oral treatment of acute IgE-mediated LPRs with the canine JAK1 inhibitor oclacitinib also featured a significant downregulation of several Th2 (*CCL17*, *IL-33*) and Th17/22 (*S100P*) markers. In addition to these, oclacitinib reduced the mRNA expression of various other immune markers in IgE-mediated LPRs at 24 h, including chemokines and chemokine receptors (e.g., *CCL19*, *CCR4*, *CXCR6*), proinflammatory and regulatory markers (e.g., *IL-18*, *LTB*, *IL-10*), and Th1 markers (e.g., *IL12RB2*). Notably, the expression of *CCL17* and its receptor, *CCR4*, was significantly downregulated by oclacitinib treatment, resembling the effects of JAK inhibitors in human lesional AD skin [16,47]. The interaction between CCL17 and CCR4 is crucial for the initial migration of Th2 cells into the dermis in inflammatory skin conditions in both humans and dogs [48,49,50,51]. Our study results suggest that, in addition to JAK-dependent cytokines, oclacitinib could modulate the gene expression of a few immune markers signaling through other pathways in acute canine IgE-mediated cutaneous reactions.

In this study, GSVA was used to compare the effects of prednisolone and oclacitinib on select AD-related immune pathways in an experimental acute IgE-mediated model [33]. The GSVA gene set enrichment method enables unsupervised assessment of pathway activity within a sample population [27]. Given the differences in the mechanisms of action of prednisolone and oclacitinib, prednisolone treatment exerted a broader immunomodulatory effect, significantly downregulating the expression of the Th1, Th2, and Th22/IL22 pathways compared to untreated IgE-mediated LPRs. In comparison, oclacitinib treatment only downregulated the Th2 and Th22/IL22 pathways in the acute IgE-mediated lesions. Although oclacitinib primarily inhibits signaling by JAK-dependent cytokines, further studies are needed to better understand the potential secondary effects of JAK inhibitors on cutaneous gene transcription in spontaneous AD patients and canine AD models. Analysis of the effect of each drug on relevant biological processes using the MetaCore platform (Process Networks) confirmed a broader and more potent downregulation of pathways related to cutaneous inflammation (e.g., lymphocyte proliferation, chemotaxis, antigen presentation) and barrier function (e.g., cell adhesion and cell–matrix interactions, extracellular matrix remodeling, connective tissue degradation) in IgE-mediated lesions treated with prednisolone compared to oclacitinib. The pathogenesis of spontaneous human and canine AD is characterized by immune dysregulation, predominantly involving activation of the Th2 pathway as well as the Th1, Th17, and Th22 pathways [1,52]. Given the importance of the Th2 immune response in AD pathogenesis, Th2 cytokines have recently emerged as targets for the development of novel therapeutics in human AD [53,54,55]. Amongst these, *IL-13* has recently been suggested as the key driver of the peripheral immune response of allergic diseases in humans [56,57]. Overexpression of *IL-13* in the skin of human AD patients is implicated in several features of AD pathogenesis, including the recruitment of inflammatory cells, disruption of the skin microbiome, decreased barrier function, and the activation of sensory nerves involved in the itch mechanism [57,58]. A recent large-scale RNA sequencing study revealed that *IL-13* was among the top upregulated Th2 markers in canine lesional and nonlesional AD skin, confirming initial RT-qPCR gene expression studies in canine AD [59,60,61,62]. In this study, intradermal injections of anti-canine IgE induced a significant increase in *IL-13* mRNA expression in LPRs 24 h post-challenge compared to healthy skin (FC = 7.3, *p* < 0.0001). While prednisolone downregulated *IL-13* expression in the IgE-mediated lesions (FC = −2.8, *p* = 0.04), oclacitinib showed no statistically significant downregulation. These differences can be attributed to the distinct mechanisms of action of each drug, with prednisolone directly regulating the transcription of immune and proinflammatory cytokines by interacting with the nuclear genome [35,36,37]. While the genomic actions of glucocorticoids on cytokines, including *IL-13*, result in a potent and broad anti-inflammatory and immunosuppressive profile, JAK inhibitors, such as oclacitinib, exert their therapeutic effects by disrupting the signaling of JAK-dependent inflammatory and pruritic cytokines [43,63,64,65].

Skin barrier impairment is a key feature of human and canine AD and has been detected at the molecular level in human patients [59,66]. Studies evaluating the effects of glucocorticoids on human and mouse skin have identified adverse effects on skin barrier integrity, including skin atrophy, increased barrier permeability, and disruptions in lipid production [67,68,69]. Short-term topical administration of clobetasol has been found to have negative effects on the stratum corneum of healthy human and mouse subjects [68]. Although visible cutaneous changes or differences in transepidermal water loss (TEWL) scores were not observed, human subjects treated with clobetasol experienced longer barrier recovery times following injury [68]. The molecular effects of glucocorticoids on the skin in human AD have often contrasted with the observed clinical outcomes. Topical administration of betamethasone valerate has been found to normalize the mRNA expression of *FLG* and *LOR* in lesional human AD skin using microarray analysis; however, there was significantly reduced expression of *IVL* and markers involved in lipid synthesis and ceramide binding [70]. Normalization of a few barrier markers (e.g., *LOR*, *KRT16*) in lesional AD skin after topical glucocorticoid treatment has also been reported in another study [41]. The effect of glucocorticoids on the skin barrier function of spontaneous canine AD patients and canine AD models remains largely unexplored. As seen in human and murine studies evaluating glucocorticoid administration on the transcriptome of healthy and AD skin, oral prednisolone treatment produced a notable modulation of several skin barrier markers in IgE-mediated lesions in healthy dogs at 24 h. In our study, we detected significant downregulation of key epidermal barrier markers *FLG*, *IVL*, *DSC1*, *CLDN2*, *CLDN11*, and *FADS1* in prednisolone-treated acute lesions. Prednisolone treatment also normalized the abnormal expression of several epidermal lipid markers affected in IgE-mediated cutaneous reactions (e.g., *FABP3*, *FABP4*, *FADS2*, *LPIN1*). Interestingly, other lipid markers, such as *ALOX12B*, *ELOVL1*, and *HMGCS1*, were significantly downregulated. JAK inhibitors have been reported to improve skin barrier function in humans [71]. Considering that glucocorticoid use can have adverse effects on the skin barrier, previous human and murine studies have suggested supplementing glucocorticoid treatment with topical application of stratum corneum physiological lipids, noting successful improvement in epidermal permeability barrier function following administration [72]. JAK inhibitors have also been reported to improve skin barrier function in human AD [71]. Abrocitinib treatment upregulated the expression of epidermal barrier products, including the tight junction markers *CLDN8* and *CLDN23* and the terminal differentiation marker *LOR*, in lesional human AD skin, suggesting an improved epidermal barrier [16]. In our study, oclacitinib treatment of IgE-mediated acute lesions significantly reduced the mRNA expression of several important skin barrier markers at 24 h, including those found in desmosomes (e.g., *DSC1*, *DSG1*, *TGM3*), tight junctions (e.g., *CLDN2*, *CLDN11*), and the lipid component of the skin barrier (e.g., *ALOX12B*, *ELOVL1*). Through their effects on other pathways, glucocorticoids and JAK inhibitors may indirectly alter the expression of important skin barrier markers in dogs with AD. Further studies evaluating barrier integrity and protein expression of these markers in canine AD patients following JAK inhibitor treatment are needed to better understand the effects of JAK inhibitors on the skin barrier in atopic dogs. Currently, the effects of JAK inhibitors and systemic and/or topical glucocorticoids on the epidermal barrier transcriptome and function in spontaneous canine AD patients remain largely unexplored, underscoring the need for further studies.

The small sample size, unpaired study design, and re-analysis of negative and positive control data may limit the findings of this study. However, as all the dogs enrolled belonged to the same colony and given the power analysis used, we believe that the results of this study provide valuable insights and validate the experimental atopic IgE-mediated acute model in dogs. While histopathological scores were not evaluated in this study, previous studies have confirmed that prednisolone effectively reduces cellular infiltrate in experimentally induced canine IgE-mediated cutaneous reactions [11]. It is important to note that, in line with previous studies, we evaluated a proactive model in which anti-allergic medications were administered before lesion induction [2,11,73]. Proactive models may differ from the therapeutic approach used in real clinical settings, where medications are given following lesion development. Despite this, our results provide a successful proof-of-concept analysis, successfully identifying and characterizing the immunomodulatory effect of distinct anti-allergic drugs on acute IgE-mediated lesions in dogs.

## 5. Conclusions

Validating experimental models that replicate the histological and molecular features of inflammatory skin diseases is crucial for assessing novel human and canine therapeutics in preclinical trials. In this study, we demonstrated differences in the immunomodulatory effects of distinct anti-allergic drugs on an experimental IgE-mediated model of acute atopic lesions in healthy dogs. As shown in in vivo studies, glucocorticoids exerted a broader and more potent immunosuppressive and anti-inflammatory effect than JAK inhibitors in this canine IgE model. Future studies should supplement transcriptomic assessment with protein expression analysis of relevant epidermal barrier proteins on this canine IgE model. While reliable antibodies for staining structural proteins in canine skin are currently limited, future advances in staining techniques would help better understand the effects of available therapeutics (e.g., JAK inhibitors, glucocorticoids, monoclonal antibodies) on the skin of canine AD patients.

## Data Availability

The original contributions presented in this study are included in the article/Supplementary Material. Further inquiries can be directed to the corresponding author.

## Supplementary Materials

The following supporting information can be downloaded at: https://www.mdpi.com/article/10.3390/vetsci13070676/s1, Supplementary Material and Methods; Supplementary Figure S1: Principal Component Analysis (PCA) plot visualizing the transcriptome profiles of the control (healthy skin, saline-injected skin, IgE lesions without drug modulation) and treatment (IgE lesions on prednisolone, IgE lesions on oclacitinib) groups 24 h post-intradermal injections. A clear separation delineated by the ellipses can be seen between the control groups. Treatment with anti-allergic drugs shifted the transcriptome profile of IgE-mediated reactions away from the untreated IgE-mediated reactions and closer to the healthy and saline control groups. Each dot represents a dog; Supplementary Figure S2: Expression of selected relevant skin barrier genes in IgE lesions without drug modulation versus healthy skin, IgE lesions on prednisolone versus IgE lesions without drug modulation, and IgE lesions on oclacitinib versus IgE lesions without drug modulation at 24 h following intradermal injections. Prednisolone exerted a stronger modulation of immune and skin barrier markers dysregulated in IgE-mediated lesions than oclacitinib. Genes are grouped by their function or family. Color corresponds to increased (red) or decreased (blue) gene expression. Statistically significant fold changes (FC) are bolded and marked with asterisks corresponding to their significance value. Significance: * *p*-adj < 0.05; ** *p*-adj < 0.01; *** *p*-adj < 0.001; **** *p*-adj < 0.000; Supplementary Table S1: Gene expression data for comparisons between healthy skin and saline-injected skin at 24 h, healthy skin and IgE lesions without drug modulation at 24 h, IgE lesions without drug modulation and IgE lesions on oclacitinib at 24 h, and IgE lesions without drug modulation and IgE lesions on prednisolone at 24 h. NA indicates that the gene was excluded from statistical analysis by the software; Supplementary Table S2: Statistically significant MetaCore process networks for downregulated DEGs (FC < −1.5 and FDR < 0.05) in treated (prednisolone, oclacitinib) versus untreated IgE lesions at 24 h post-injection and upregulated DEGs (FC > 1.5 and FDR < 0.05) in untreated IgE lesions versus healthy skin at 24 h post-injection.
