# Supplementary figures and images for: Transcriptomic Effects of Oclacitinib and Prednisolone in an Acute IgE-Mediated Experimental Model of Canine Atopic Dermatitis
Source: Vet Sci. 2026 Jul 13;13(7):676. doi: 10.3390/vetsci13070676 (PMC13431516; doi:10.3390/vetsci13070676)

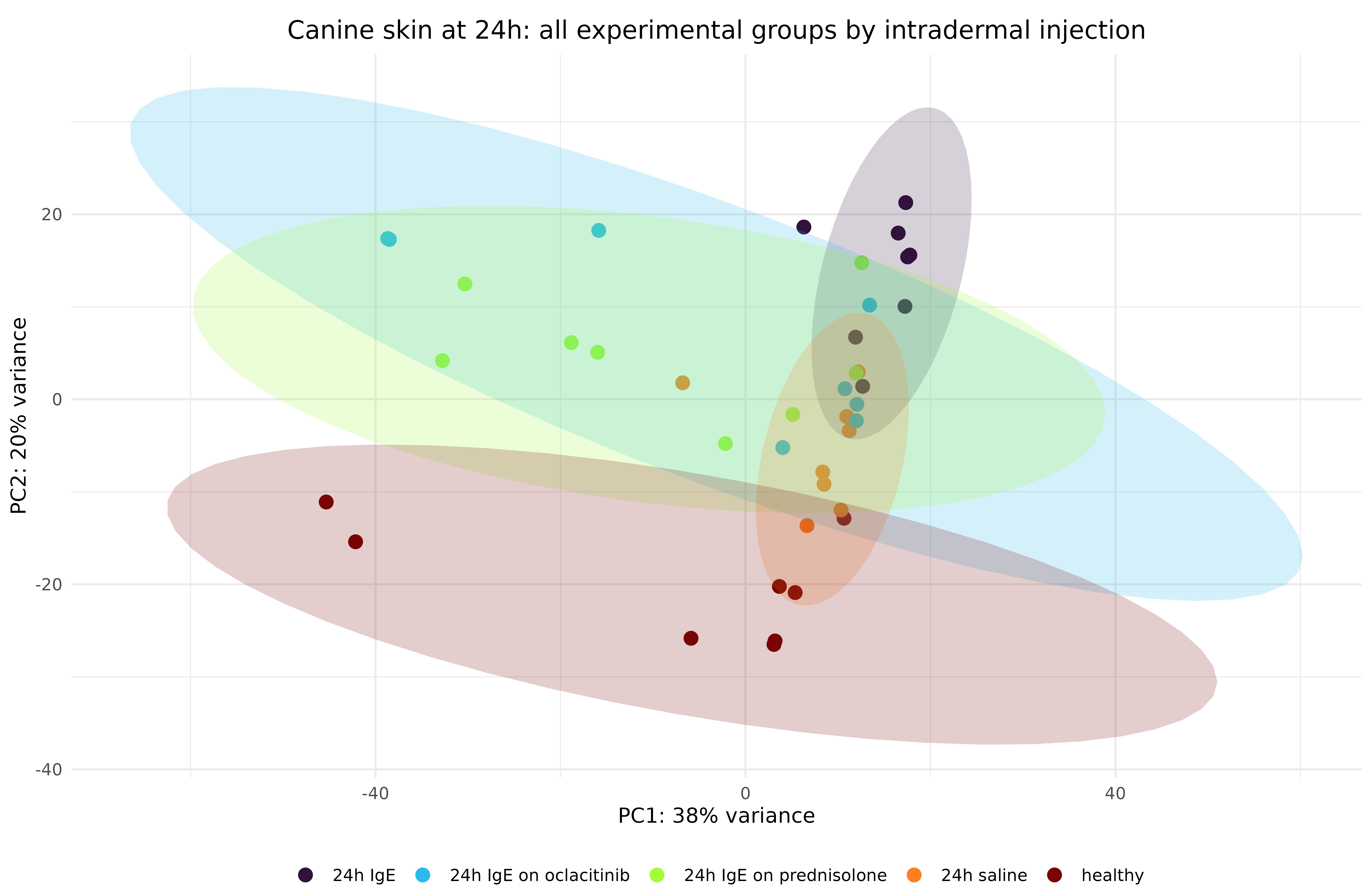

Supplement: Supplementary file 1 [file vetsci-13-00676-s001.zip › Supplementary Figure S1_PCA.tiff]

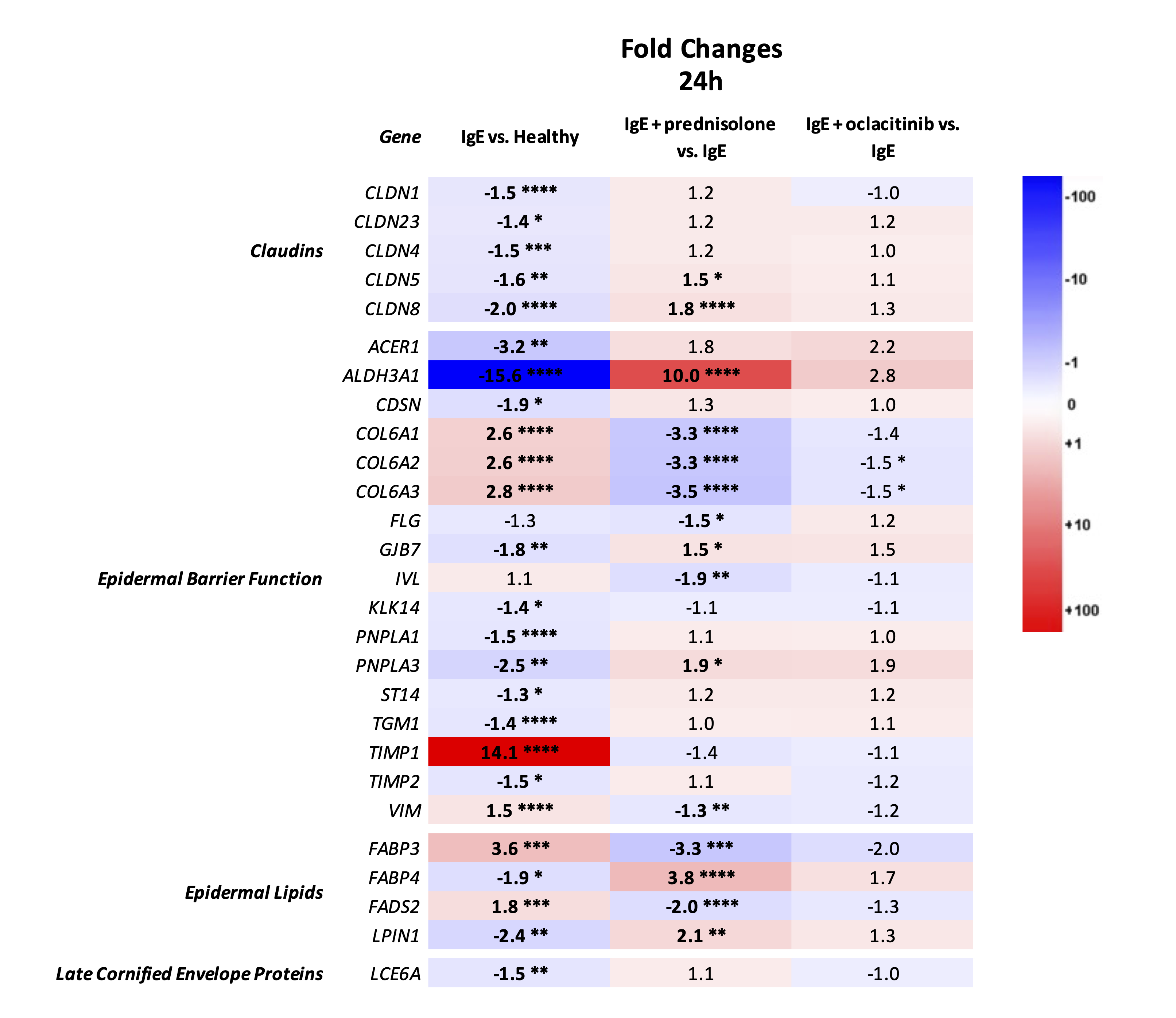

Supplement: Supplementary file 1 [file vetsci-13-00676-s001.zip › Supplementary Figure S2.tiff]
